# Social-emotional learning in physical education classes at elementary schools

**DOI:** 10.3389/fpsyg.2025.1499240

**Published:** 2025-04-04

**Authors:** Mahmood Sindiani, Hadas Brodie Schroeder, Ayelet Dunsky

**Affiliations:** The Levinsky-Wingate Academic College, Wingate Institute, Netanya, Israel

**Keywords:** teamwork, self-awareness, creative thinking, reflection, physical education, social-emotional learning

## Abstract

**Introduction:**

The study investigates the impact of a social–emotional learning (SEL) intervention program implemented during physical education classes on elementary school students. The objective was to examine the development of social–emotional skills, focusing on teamwork, self-awareness, and creative thinking.

**Methods:**

The intervention, grounded in the SEL model, spans three 45-min sessions and involves 260 students (grades 4–6) from two schools in northern Israel. The experimental group (eight classes) participated in the SEL program, while the control group (three) received a standard 45-min physical education class. The research methodology was based on a *convergent* mixed model combining quantitative observations and qualitative interviews with physical education teachers.

**Results:**

Significant improvements were noted in the experimental group across all evaluated skills, indicating a positive impact on personal and interpersonal social development. The findings extend beyond students favoring sports, benefiting even those who previously avoided physical education classes. Notably, the intervention enhanced reflective skills in both students and teachers. The qualitative insights highlight the program’s positive impact on creative thinking, an aspect that had not been previously observed.

**Discussion:**

The study emphasizes the potential of integrating SEL into physical education to foster social–emotional skills and overall well-being. It underscores the importance of creating a positive and emotionally supportive learning environment, contributing significantly to students’ holistic development. Overall, this study provides valuable insights into leveraging physical education classes for intentional SEL interventions, offering a promising avenue for promoting social–emotional growth among elementary school students.

## Introduction

In today’s era, education systems are expected to instill academic literacy in students, especially in core subjects, providing them with valuable and necessary skills for the present and the future. For example, academic literacy could enhance their ability as adults to work with individuals from different backgrounds by applying social and emotional skills, demonstrating healthy behaviors, and acting responsibly and respectfully toward others (Greenberg et al., 2005). As such, one key component in today’s education processes entails cultivating social–emotional learning (SEL) – especially considering the accelerated technological developments and cultural and social changes that characterize the 21st century. Such skills are reflected through care and concern for others, resolving problems as they arise, improved academic achievements, and decreased behavioral issues and emotional distress (Benbenishti and Friedman, 2020; Zins et al., 2004).

Such abilities can be enhanced over time in behaviors shaped by the individual’s internal values while seeing the other and accepting responsibility for one’s personal choices and behaviors (Benbenishti and Friedman, 2020). Associations have been seen between these skills and the individual’s personal, social, and occupational success. According to the Collaborative for Academic, Social, and Emotional Learning Model, teachers must help students build and strengthen five skill sets: self-awareness, self-management, social awareness, relationship management, and informed decision-making (Yang et al., 2018).

By the fourth grade, students should be able to apply self-awareness, identify the feelings, strengths and weaknesses of others, and comprehend connections between emotions, sensations, and behaviors – while regulating them and demonstrating restraint and delayed gratification. They should also know how to set personal and social goals and adapt to change. Regarding social awareness and interpersonal skills, students at this age should be able to exhibit empathy toward others, listen to different opinions, and work as part of a team while recognizing the contribution of their peers to the group’s success. Finally, they should also be able to recognize and avoid dangerous situations, resist negative social pressure, and contribute to a positive atmosphere in the classroom (SEL.IL Center at the Reichman University and the Tel Aviv-Yafo Municipality Director of Education, 2022).

The underlying premise of the current study is that the school setting serves as a valuable training ground for enhancing social–emotional development in students and could be leveraged to enhance their mental well-being, cognitive development, and knowledge. Therefore, SEL skills should be cultivated, imparted, and integrated into all lessons. Based on this suggestion, this study aimed to explore the potential implementation of SEL elements in physical education classes for elementary school children.

In addition to creating an environment that nurtures SEL in all classes, SEL-dedicated lessons should become an integral part of the school curricula. Doing so will enable students to acquire emotional and social skills while developing positive self-perceptions, values, and worldviews. Moreover, teachers should convey how such skills can be transferred to additional topics that comprise the learners’ academic, social, and personal lives (Benbenishti and Friedman, 2020).

The literature indicates that explicit intervention programs that effectively promote SEL in students are those that meet the SAFE criteria: (S) *Sequenced* training and learning in a gradual and continuous, step-by-step process, (A) *Active* programs that combine both learning and practicing of SEL skills, (F) *Focused* programs that devote time to the development of each newly acquired SEL characteristics, and (E) *Explicit* programs that define the social–emotional skills that are being taught and addressed (Gordon, 2021; Frazier et al., 2021). Indeed, interventions that are effective in instilling SEL skills are structured as a gradual, continuous, and step-by-step process; entail active learning, including the practicing of the learned skills and elements; focus on the developing of each of these skills; and explicitly define those SEL aspects that it aims to develop. However, SEL must be conducted in line with the students’ chronological age and developmental stage, and concerning the needs or composition of the class, school, or time, such as during times of emergencies, in heterogenous or special needs classrooms that entail inclusion and integration, and the school’s educational outlook (Benbenishti and Friedman, 2020).

### SEL in physical education classes

While SEL can and should be incorporated into most subjects, physical education (PE) lessons could be especially beneficial, as PE activities are often based on teamwork and other social relationships. PE classes also provide a setting for promoting intense emotions, nurturing a positive environment for promoting SEL (Escartí et al., 2018; Gagnon, 2016), and creating positive student experiences (Dyson et al., 2021). Yet, while PE classes provide an excellent setting for exposing students to personal and group processes, they may not be adequately leveraged for this purpose (Richards et al., 2019). Indeed, if PE teachers do not know how to integrate SEL into the curriculum optimally, they may overlook this substantial component (Hughes, 2020). In turn, their classes will enhance sport-related skills through moderate-to-high-intensity physical activity. While encouraging students to lead active lives is essential (SHAPE America – Society of Health and Physical Educators America, 2019), the invaluable nurturing of SEL elements will most likely be ignored.

With the correct design of PE classes, teachers can enhance skills focusing on students’ personal and social responsibilities, such as sportsmanship, cooperation, group formation, and leadership (Ciotto and Gagnon, 2018). According to the Israel Ministry of Education, developing SEL skills aligns with PE classes’ goals, which relate to three core issues: maintaining rules and fairness, taking personal and shared responsibility, and cooperating. More specifically, these goals can be achieved while focusing on SEL skills. For example, observing the rules during a game allows children to understand how rules affect relationships within a group of individuals who conduct themselves as a society or maintain fairness, which requires the consideration of others – including their strengths and weaknesses (Barney et al., 2021).

The skill of taking responsibility should be taught gradually and in line with the students’ level of independence, as this ability requires both a theoretical and a practical understanding of what taking responsibility means. PE teachers at elementary school could first ensure that the students arrive at the correct classroom and at the right time, and later teach them how to perform warm-up exercises before the teacher arrives – a skill that requires students to make decisions in a group setting, address safety-related issues, and be familiar with the warm-up exercises. Moreover, the teacher should trust that the students can act independently and take responsibility while conveying this to them. Finally, allowing children to take responsibility requires a relatively unrestricted framework. Nevertheless, the more open the framework, the more clarity it requires regarding what is permitted or prohibited (Hughes, 2020).

Numerous situations in elementary schools require rules and fairness, individual and shared responsibility, and cooperation. In ball games, for example, beating the other team requires cooperation between the team members, who must also be familiar with the game’s rules and each player’s role on the field. The players must take responsibility for fulfilling their roles while helping others fulfill theirs. Such cooperation can be achieved when students strive to achieve the same common goals through mutual help and interdependence (Hughes, 2020).

Next, requiring children to follow the rules, take responsibility, and cooperate during PE classes – and with adult mediation – could help them apply these SEL skills to additional settings, such as other lessons and social and familial activities. However, as in any such activity in the classroom, the teacher must explain how leadership dynamics develop within a group, focusing on the emergence of subgroups who may or may not oppose the leader. Finally, to sustain collaborations and teamwork, teachers should provide students with coping strategies and mechanisms for dealing with changing circumstances or dialogs that could lead to disagreements, arguments, and even power struggles (Ciotto and Gagnon, 2018). Teaching elementary school students those core elements would prepare them for higher socio-emotional skills during their experiences at higher grades (i.e., cooperation in a group, providing help and looking after colleagues, cultivating patience and tolerance toward others while understanding the difference in abilities, and developing self-confidence in movements and positive attitudes toward movement) (Ministry of Education, Israel).

### The teaching personal and social responsibility model

One model that could be leveraged for implementing SEL in PE classes is the Teaching Personal and Social Responsibility (TPSR), which increasingly emphasizes the learners’ need to take responsibility over time. Hellison (2011) presents the TPSR as a structured model comprising progressive developmental stages that provide students with the empowerment and encouragement to take responsibility for their actions while considering the emotions of others. Moreover, by focusing on values such as effort, choice, and respect, this model enhances the personal and social well-being of the learners (Hellison, 2011). Empirical studies have validated its effectiveness in PE interventions (Escartí et al., 2018; Pozo et al., 2018), emphasizing its structured nature.

According to the TPSR model, activities and goals should align with the learners’ emotional development. In the first *egocentric* stage (ages 6–8 years), when children still lack self-control and the ability to cooperate with others, teachers should first encourage them to participate in group activities and then encourage them to respect the rights and emotions of others through self-control and peaceful problem-solving. As learners progress to *the second respect and responsibility* stage (ages 9–11), teachers should nurture the children’s ability to respect the rights and emotions of others while interacting more harmoniously with their peers. Next, in the third *effort and cooperation* stage (ages 12–14), teachers should enhance their students’ sense of commitment, effort, and cooperation while instilling a sense of responsibility and dedication. As students advance to the fourth *self-direction* stage (ages 15–17), the emphasis turns to self-management within structured tasks, where they are expected to take greater personal responsibility for their actions and progress. In the fifth stage*, Helping Others and Leadership* (ages 18–20), teachers should encourage students to display leadership qualities and help others while fostering their sensitivity and concern for the well-being of their peers. This stage aims to develop both personal and social responsibility. In the sixth and final stage, *transfer and application* (ages 21 and upwards), teachers should create opportunities for students to apply the skills and values acquired in the classroom to other real-life contexts beyond the school environment. This developmental approach reflects a comprehensive strategy for educators to nurture their students’ emotional growth, transitioning from basic cooperation skills to the more advanced social and personal skills of taking responsibility, ultimately preparing them for success in various life situations (Hellison, 2011).

Implementing the TPSR model in PE classes has significantly improved SEL skills in children and adolescents (Hughes, 2020). Studies indicate the effectiveness of applying SEL programs in PE classes in secondary school students (e.g., Akelaitis and Malinauskas, 2016; Bartlett, 2019; Escartí et al., 2018), and their positive outcomes on teenagers’ social–emotional skills (Koutelidas, 2022; Hellison 2010; Hughes, 2020; Pozo et al., 2018). However, there is a gap in the literature regarding implementing the TPSR model in physical education classes in elementary schools. To close this gap and offer new insights into SEL interventions, the participants in this study were *primary* school students, grades 4–6, when learners tend to exhibit lower levels of cooperation (Koutelidas, 2022; Hellison 2010; Hughes, 2020; Pozo et al., 2018). Moreover, elementary school students are still developing their social and emotional abilities and habits that could influence their interactions and relationships with others (Maglica et al., 2020).

As such, this study aimed to examine the feasibility of implementing an SEL model within PE classes in elementary schools while assessing the impact of this intervention on children’s social–emotional skills. The study also aimed to provide directions for the participants’ social–emotional development at this crucial timepoint for fostering positive behaviors and resolving cooperation-related difficulties (Koutelidas, 2022; Pozo et al., 2018; Richards et al., 2019). Based on the literature review presented above, we hypothesized that elementary school children will demonstrate significantly improved social–emotional skills following the implementation of this SEL intervention program.

## Methodology

### Study population

This study used convenience sampling because of logistical constraints and school accessibility. While this limits generalizability, it enables an in-depth analysis within a controlled setting. Inclusion criteria required students to participate actively in PE classes, while exclusion criteria included those with medical conditions that prevent physical activity. The study included 260 students, grades 4–6 (ages approximately 9–12), from two elementary schools in northern Israel. Data collection took place during the second half of the school year, after students had participated in approximately 15 PE lessons together. The experimental group comprised 185 students (96 females) from eight classes who underwent an SEL training intervention during three modified 45-min PE lessons. The control group comprised 75 students (36 females), from three different classes who participated in one standard 45-min PE lesson. All participants were from mainstream education classes. The study also included interviews with three PE teachers with more than ten years of experience in the field who conducted the intervention program. The study’s sample size was determined based on previous SEL research in PE settings, ensuring statistical power for detecting meaningful differences.

### Research tool

This study employed a convergent mixed methods design in which quantitative and qualitative data were collected simultaneously and analyzed to understand SEL development comprehensively. The rationale for this design was to ensure that both statistical outcomes and teacher insights contributed to interpreting the intervention’s effectiveness.

*In the quantitative* part of the study, observations of the classes were conducted to evaluate the developmental stage of the students’ social–emotional skills through observation of the skill assessment indicators during activity (see Supplementary material). Each lesson was divided into several sections, each targeting a different skill. While the intervention aimed to develop multiple social–emotional competencies, the three skills measured in the quantitative study—teamwork, self-awareness, and creative thinking—were chosen based on their direct observability in structured PE settings. Other competencies, such as interpersonal inclusion, were more difficult to quantify through observation alone and were, therefore, primarily examined through a qualitative analysis. Ten items were assessed throughout the study based on the following three skills. First, from a social aspect, four items of *teamwork skills* were examined, including (a) non-judgmental climate and discourse culture, (b) responding to conflicts between group members, (c) punctuality and following instructions, and (4) contributing to the team’s success). Next, from an emotional aspect, four items of *self-awareness* were examined, including (a) *persistence with challenges*, (b) internal control focus (reflective ability), (c) perseverance with difficulties and failures, and (d) *proactivity*. Finally, from a generic-cognitive aspect, two items of *creative thinking skills* were examined, including (a) problem solving, and (b) acquisition of strategies and skills. In each lesson, the researcher/observer was instructed to rate the degree of each item on a Likert-like scale, from 1 (just beginning to embark on the journey) to 4 (have reached the destination).

In the *qualitative section* of this study, three PE teachers took part in in-depth semi-structured interviews to assess the intervention plan from their professional perspective (see Supplementary material for sample questions used in the interviews). One researcher conducted the interviews to minimize interviewer bias and enhance analytical rigor, while two independent researchers performed the qualitative data analysis. This methodological approach ensured a more objective interpretation of the data.

### Research procedure

First, a preliminary meeting was held with the three participating PE teachers to present the research objectives and intervention plan. They were instructed to only provide the participants with instructions, not to direct or intervene during an activity. The researchers then conducted observations during all PE lessons (24 modified lessons and three regular lessons in total). In these classes, the students were informed that a PE teacher (i.e., the researcher) would observe their lesson, yet no other information was provided.

Based on the TPSR model, the intervention program was implemented through recreational and group games. Following is a description of the activities and their related SEL skills that were examined during the observations, based on the developmental stages presented above:

For the first stage, *egocentric*, the teacher initiates games requiring cooperation to achieve a shared goal. For example, the teacher divides the class into four groups and gives each group a soft ball. The group must then work together to eliminate members of the other teams while saving their team members with the ball. The guidelines presented by the teacher encourage cooperation, as the students are urged to act for the good of their team rather than for their own benefit.

For the second stage*, respect and responsibility*, the teacher presents role-play scenarios that require the children to consider the rights and feelings of others. Students then perform in line with the scenarios, including trading roles, sharing resources, and amicably settling disputes. For example, the teacher divides the class into two groups and gives each group 12 hoops and two balls. While one group is asked to build a “galaxy” using a four-hoop structure, the other group attempts to ruin their work by throwing their balls at the newly built structures. Each member of the two teams is given a specific role, such as “builder” or “defender,” taking responsibility and behaving respectfully while complying with the game’s rules.

For the third stage*, effort and cooperation*, the teacher presents group tasks. For example, after dividing the class into groups, the teacher may give each group a set of cones organized like a mobile phone keypad (i.e., 123, 456, 789). The teacher calls out different numbers after each student is allocated a number between 1 and 9 (the groups are allocated the exact numbers). Students whose number is called out must move as quickly as possible to the area marked by the cones, yet in the sequence of the numbers. The first group that stands in the correct position wins. Later, this task can be performed using letters instead of numbers.

For the fourth stage*, self-direction,* the teacher first helps the children set goals for themselves in line with their capabilities and even aspirations, such as submitting their homework on time. Next, the teacher invites the pupils to actively engage in discussions at the end of both the game and the lesson, with an emphasis on their personal growth and development during the related exercise.

For the fifth stage*, helping others and leadership*, students are offered opportunities for exhibiting their leadership qualities while supporting their peers. For example, the teacher can instruct the teams to move from one side of the room to the other as quickly as possible using hoops. Five hoops are placed on the ground in a line, with one student standing inside each hoop. The last hoop is then moved to the front of the line, through teamwork, and so forth – until the team reaches the other side of the room. Additional challenges can be introduced once the game is understood, such as only using three hoops or completing the task within a given timeframe. While assuming leadership throughout the activity, each student must make decisions, lead their peers, and communicate clearly. This activity encourages children to support and assist one another while developing a sense of unity and empathy. After the activity, the teacher conducts a discussion where the students can speak of their experiences and the value of leadership, teamwork, and support when working together to achieve a shared objective.

For the sixth and final stage, *transfer and application development*, the tasks entail real-world problem-solving and a community fitness challenge. Doing so enables the students to apply their acquired social–emotional skills in a practical setting while promoting physical health. For example, the teacher may divide the class into teams and then instruct them to develop and implement a fitness challenge for the school neighborhood, such as creating an obstacle course. The teams organize the event, including choosing a location, determining the route, and selecting the activities. To guarantee the successful organization of the event, students must effectively communicate with one another, work together to make choices, and exhibit leadership traits. The students then conduct the event, possibly followed by a discussion with the teacher, to enable them to reflect on their teamwork, leadership, communications, and problem-solving abilities.

### Ethics statement

According to the permit issued by the ethics committee, the participants' parents or legal guardians did not need to provide written informed consent for this study. The permit specifies that written informed consent is not obligatory as long as the observations are recorded in writing only and without recording any identifying details. The ethical approval for this study was obtained from the Chief Scientist’s Office at the Ministry of Education, and all procedures were conducted under their guidelines and regulations.

### Data analysis

To test the factor reliability of the observational research tool applied in this study, Cronbach alpha analysis was conducted; to examine the development of the participants’ socio-emotional skills between sessions, Friedman (a-parametric) χ2 tests were conducted, as variable distribution was non-normal. Wilcoxon signed-rank tests were used to compare paired observations over time within the experimental group. The Mann–Whitney U test for independent samples was used to analyze differences between experimental and control groups.

The qualitative data were analyzed using a grounded theory approach (Shkedi, 2003). Transcripts were coded inductively to identify emerging themes. A triangulation process was employed to ensure reliability, with two independent researchers coding the data and resolving discrepancies through discussion.

## Results

Strong factor reliability was seen in three measurable skills with Cronbach’s *α* = 0.97 for *teamwork*, 0.98 for self-awareness, and 0.98 for *creative thinking*. Friedman test results indicated significant improvements for all stages of the intervention in the three examined skills: *teamwork* [χ^2^_(2)_ = 156.867, *p* < 0.001]; *self-awareness* [χ^2^_(2)_ = 154.226, *p* < 0.001]; and *creative thinking* [χ^2^_(2)_ = 125.511, *p* < 0.001]. Based on assessments from the first session to the second, and from the second session to the third, Wilcoxon tests presented significant differences between measurements in the experimental group: *Teamwork* [(*T* = 2,850, *Z* = −7.594, *p* < 0.001) and (*T* = 465, *Z* = −4.820, *p* < 0.001), respectively]; *self-awareness* [(*T* = 2,775, *Z* = −7.605, *p* < 0.001) and (*T* = 325, *Z* = −4.435, *p* < 0.001), respectively]; and *creative thinking* [(*T* = 1770, *Z* = −7.320, *p* < 0.001) and (*T* = 171, *Z* = −4.146, *p* < 0.001), respectively].

**Figure 1:**
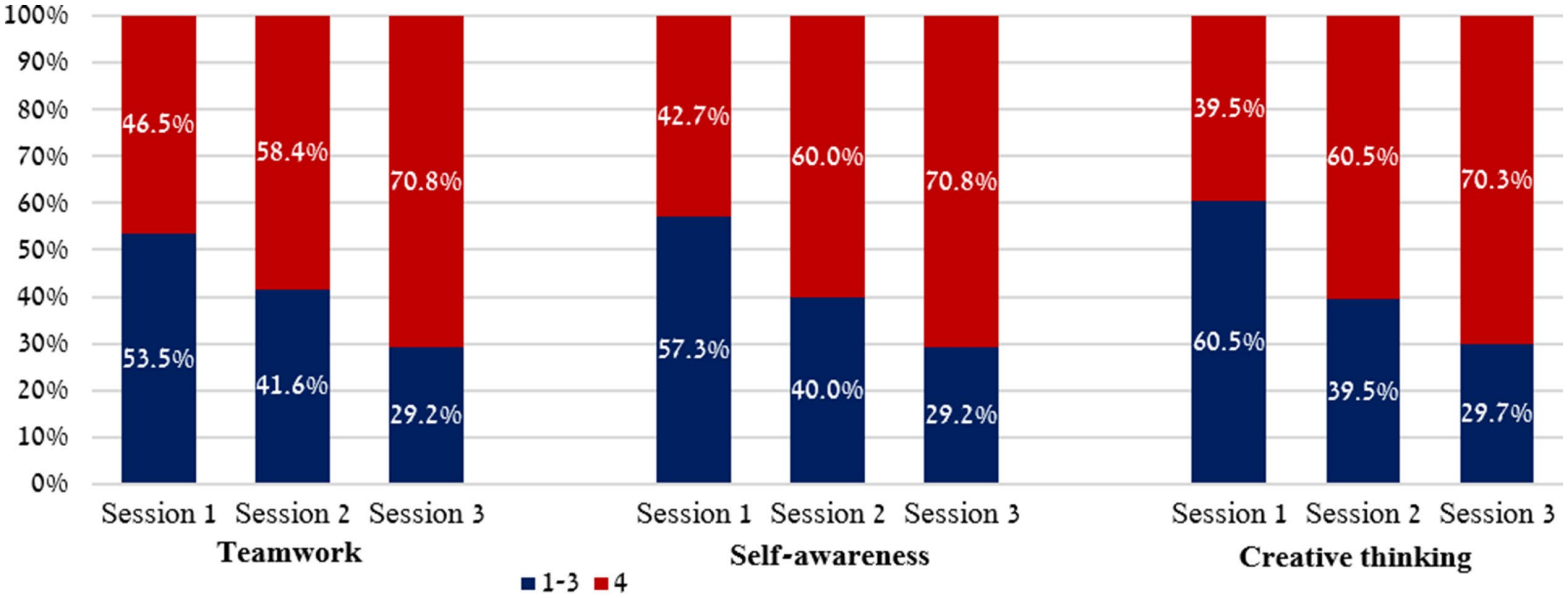
Percentage of students in the experimental group achieving different levels of SEL skills across three intervention sessions.

As seen in Figure 1, while only 46.5% of the students achieved the maximum mean score for *teamwork* in the first session, this number increased to 58.4% in the second session, and to 70.8% in total. For *self-awareness*, while only 42.7% of the students achieved the maximum score in the first session, this number increased to 60% in the second session, and to 70.8% in the third session. Finally, for *creative thinking*, while only 39.5% of the students achieved the maximum 4-point mean score in the first session, this number increased to 60.5% in the following session and to 70.3% in the third session – indicating the greatest improvement of the three skills.

Finally, when comparing between the final session of the experimental group and the control group, significant differences were seen in all three skills: *teamwork* [MW = 1041.500, *Z* = −11.594, *p* < 0.001]; *self-awareness* [MW = 1143.500, *Z* = −11.440, *p* < 0.001]; and *creative thinking* [MW = 1354.500, *Z* = −11.200, *p* < 0.001], thereby indicating significantly higher scores among the experimental group than the control group (see Table 1 for the Wilcoxon paired tests).

**Table 1:** The Wilcoxon paired tests include effect size computations (r) to assess the strength of observed differences.

Wilcoxon tests	1 vs. 2	2 vs. 3
Teamwork (r)	−0.5583	−0.3544
self-awareness (r)	−0.5591	−0.3261
creative thinking (r)	−0.538	−0.305

Mann–Whitney U test	final session
Teamwork	−0.719
self-awareness	−0.709
creative thinking	−0.695

Additionally, Mann–Whitney U test results rank-biserial correlation coefficients (r) to measure effect sizes.

Based on the grounded theory methodology, the interviews were analyzed through open coding (Shkedi, 2003), leading to the emergence of the following five themes, presented here by significance (i.e., from the highest to the lowest number of times the skill was mentioned by the interviewees): (1) social skill of cooperation; (2) creative thinking; (3) reflective abilities; (4) empathy; and (5) interpersonal inclusion (see Table 2 for summary of qualitative findings from teachers interview).

**Table 2:** Summary of qualitative findings from teacher interviews*.

Theme	Description	Supporting quotes from teachers
Cooperation	Students demonstrated increased teamwork and collaboration during the intervention. They understood that success depends on collective effort rather than individual performance.	*“Helping each other, talking to one another… understanding the power of the group as a group.”* (Teacher 1)
Creative thinking	Participants showed an ability to think outside the box, find new strategies, and approach challenges in innovative ways.	*“The students really surprised me. They performed the tasks even more successfully than I had hoped.”* (Teacher 3)
Reflective abilities	Students developed skills in self-reflection, recognizing their learning process and ways to improve their performance. Teachers also reflected on their own instructional methods.	*“The students were able to raise important points for discussion… They learned from their experience and improved in the next sessions.”* (Teacher 2)
Empathy and interpersonal inclusion	Some students exhibited empathy by considering their peers’ feelings and supporting one another during group activities. However, this was less emphasized compared to other skills.	*“Some students showed an interest in how others were feeling, which was really great to see.”* (Teacher 3)
	The intervention helped students, particularly those who typically struggle in PE classes, feel more included and engaged. The structured group activities allowed them to contribute meaningfully.	*“Students who usually do not enjoy PE realized they had a role in the group, which made them feel more confident.”* (Teacher 1)

*The table below presents the order of the times that the teachers mentioned the skills from the highest to the lowest.

### Skill 1: cooperation

The students’ willingness to cooperate with one another was mentioned a total of thirteen times during the three interviews. As stated by interviewee 1, “Helping each other, talking to one another. With the aim of finding a solution through teamwork and cooperation. Understanding the power of the group as a group.” Similarly, Interviewee 3 said, “There was notable collaboration between the students.” Finally, interviewee 3 stated:

The social skill of cooperation is reflected in the understanding that tasks cannot be completed without the participation of all group members. The understanding that success is the group’s, not the individuals’. Everyone had to participate in order to succeed… This helped the students realize that PE does not have to be a kind of ‘punishment.’ It can be a fun class that they look forward to.

Teachers’ observations reinforced the quantitative findings by highlighting significant improvements in teamwork, self-awareness, and creative thinking. Additionally, reflective skills and interpersonal empathy emerged as further benefits, even though they were not directly quantified. The teachers also spoke of the children’s fear of failure that was felt in the first class – yet that gradually disappeared in the next two classes, as the children began to understand that success is a group effort. As Interview 1 explained, “At first, they may have worried about failing, but overtime, they began to realize that it’s OK to make mistakes, learn from them, correct them, and the succeed.” Finally, the teachers expressed how the group tasks strengthened the social status of those students who are not always chosen by their peers, providing them with an opportunity to improve their social skills through these tasks.

### Skill 2: creative thinking

The second most mentioned skill, i.e., creative thinking, appeared nine times throughout the interviews. For example, thinking outside the box as a means for solving a task was noted by the teachers as both surprising and as one that improved from task to task and from session to session. As stated by Interviewee 3: “I was delighted. The students really surprised me. They performed the tasks even more successfully than I’d even hoped.” Interviewee 1 also spoke along these lines, saying, “I think it’s creative thinking, thinking about subjects in new ways. And the students were open to new ideas and thought outside the box.”

### Skill 3: reflective skills

The third most commonly mentioned skill, coded five times, is the ability to reflect, with students recognizing the processes they had gone through while carrying out the task, and offering feedback for improving their future performance. Here too, the teachers were pleasantly surprised by the level of execution of the skill, as seen in the following quotes: “The students were able to raise important points for discussion, which surprised me. I was delighted to hear them speak with confidence, answer questions that show their understanding of the process, learn from their experience, and perform better next time” (Interviewee 2). The teachers also spoke of how the students were able to discuss issues regarding their performance, consider changes that they need to apply to succeed, and decipher new situations. With this skill, the teachers reported being pleasantly surprised by the students, who when given the opportunity to reflect on the process in a structured manner were able to evaluate their current performance and think about how to improve their future performance.

The teachers also self-reflected on the new teaching approach to which they were exposed via the intervention program. For example, they explained that prior to the intervention, they would control the games and give instructions with the sole focus on PE aspects. The SEL intervention program, however, required them to emphasize social–emotional skills that they would usually overlook. With the program requiring students to act with greater freedom, and the teachers to relinquish much of their control – the students took much greater responsibility than usual, which pleasantly surprised the teachers. As explained by Interviewee 2, “At first I was worried that they would use it [their freedom] negatively place, make jokes, or not cooperate. But in fact I was really happy to watch them. It was amazing. They were really interested, they were learning, enjoying themselves, playing, and cooperating in an amazing way.” Interviewee 3 also spoke along these lines, saying, “Usually in my classes I’m the one who controls the class atmosphere. I usually give the students much less freedom. Here in the classes I really liked the freedom I gave them, and I saw how it created a really pleasant atmosphere. I really liked it.” Interviewee 1 even said:

The program is especially great if you are talking about seasoned teachers who are fixed in their ways, because the program requires taking a different direction, a different line of thought, alternative options. The classes also gave the students a place, a kind of responsibility, which is a very important thing, to change existing habits or develop new ones that can help them. It was a huge responsibility to be close to the guidelines and it made it all the more difficult. I had to be fully focused all the time [he says with a smile].

### Skill 4: interpersonal empathy

The fourth and final skill that emerged from the interviews, coded twice, relates to interpersonal empathy. It seems that this skill was developed to a lesser extent than the other three capabilities during the intervention program, and was only mentioned by the teachers in the context of helping others and attempting to improve the group’s performance. As stated by Interviewee 3, “Another thing that stood out is that some students exhibited empathy and showed an interest in other people’s feelings.” The teachers also mentioned pleasant and respectful discourse – where the children listened to one another – yet without mentioning the terms *empathy* and *identifying with other people’s feelings*. Nuruting this skill may require additional elements in the intervention program, such as a longer program, increased practice, or additional mediation. As such, future research should specifically address the development of such skills in children through SEL interventions in PE lessons.

## Discussion

This study aimed to assess the feasibility of implementing an SEL intervention program in elementary-school PE classes. The results reveal significantly higher scores for all examined SEL components and skills in the experimental group compared to the control group.

From a social aspect, improvements were seen in *teamwork* in all four parameters. This finding is in line with Goudas and Magotsiou (2009), who found a significant increase in the social skill scores of an experimental group following an SEL intervention, based on cooperating skills, empathy, and quick-temperedness. Although their intervention lasted 5 weeks (compared to the three-week intervention in this study), the improvements found in both studies among the experimental groups could probably be addressed as the outcome of including cooperative learning activities in the lessons, such as dividing the students into small groups, promoting feedback, encouraging support for other group members, and suggesting alternative roles while playing games.

From an *emotional aspect*, a significant improvement was seen in all four items of self-awareness, thereby implying that in addition to working on developing physical skills, students could improve their ability to deal with challenges, overcome failure and disappointment, and reflect on such situations. It seems that the questions that the teachers were instructed to ask during the intervention program (such as, “What did you learn during the ball game?” or “How do you think you behave when someone hits you with the ball?”) helped the participants recognize and assess these situations. This study’s findings align with Malinauskas and Malinauskiene (2021), who found that the participants exhibited improved self-control after participating in an SEL training program that included 48 15-min sessions. In the current study, although the participants only took part in three PE classes, each class was substantial, lasting 45 min. Moreover, as each lesson was divided into several sections, the intervention was able to target a range of social–emotional skills.

From a generic-cognitive aspect, the significant improvement seen in both examined items is in line with Gorucu (2016), who found improved self-control in secondary school students following a 10-week intervention.

Combining the qualitative and quantitative findings offers a more thorough understanding of SEL development. While statistical results confirmed significant improvements in teamwork, self-awareness, and creative thinking, the qualitative interviews provided further insights into students’ reflective abilities and empathy. In the qualitative interviews, the teachers reported on improving their students’ performance from session to session and in the reflective dialogs between the group members with the teacher’s guidance. The teachers also testified that as educators, and since the interviews required them to reflect on their classes, they observed their teaching and recognized how giving their students autonomy leads to surprisingly positive results – with the students taking responsibility for their actions and goals. As such, the SEL implementation feasibility plan implemented in this study created an opportunity for the teachers themselves to improve their teaching processes.

An additional point raised in the teachers’ interviews was that the intervention program, which required the students to work together in groups, positively affected those students who usually do not enjoy sports and even refrain from participating in PE classes if possible. Nevertheless, during the SEL intervention program, these same students recognized that they were part of a group and that, while their contributions affected the success or failure of the group as a whole, they were not alone in these tasks. This seemed to have instilled an enhanced sense of confidence and competence as they began to enjoy participating actively in the lessons. Finally, the teachers also reported that the students’ creative thinking skills had developed during the intervention program. Compared to regular PE classes, the teachers’ minimal instruction and intervention provided the students with increased opportunities for reflection and the desire to succeed as a group in future sessions. Finally, it is important to note that interpersonal empathy was the least mentioned skill in the interviews. This finding aligns with Miller and Hoffman (2009), who suggested that developing empathy requires long periods and repeated experiences, especially in competitive sports, where competitiveness may exceed empathy.

Our findings align with recent studies demonstrating the effectiveness of SEL interventions in physical education. For example, studies by Escartí et al. (2018) and Pozo et al. (2018) support the positive impact of SEL programs on students’ social–emotional competencies. These results reinforce the relevance of structured SEL programs in school settings. The significant changes seen in experimental groups following SEL intervention programs in PE lessons indicate the positive impact of SEL training on social–emotional competencies and general behaviors. As seen in this study, PE classes can also support and enhance various non-physical skills in addition to helping students prepare for long-term healthy lives through cardiovascular endurance and muscular strength. When doing so, it is important for PE teachers to emphasize the five core competencies of self-awareness, self-management, social awareness, relationship management, and informed decision-making (Yang et al., 2018). They can do so in almost any PE lesson. Moreover, according to the Positive Youth Development Approach, PE classes can enhance developmental abilities and health-related outcomes when targeting practical social-contextual features such as skill-building activities and supportive relationships (Weiss, 2011).

## Limitations and future research

The findings of this study provide an important contribution to the literature on SEL in elementary-school PE lessons. However, several research limitations should be addressed. First, using a convenient sampling method restricts the generalizability of the findings. Future research should aim for randomized sampling to improve external validity. Next, only two elementary schools were included in the study, with just three PE lessons. As such, generalization of the findings should be made with caution. Future research could benefit from including many elementary schools, preferably from larger towns, and several more classes using SEL elements. In addition, only students from mainstream education were included in the study. However, classes and schools often include children with unique characteristics and thus may have special education needs. Moreover, SEL retention and transfer were not measured in this study. Therefore, future studies should examine these skills in additional frameworks, such as in other non-PE classes and outside of school. At the same time, the current study’s results align with Korem (2021), who states that “teachers skip strategies for developing competence and go directly to encouraging social interactions.” In other words, while PE teachers may encourage their young students to participate in constructive social interactions through a range of activities and shared goals, they do not instill social skills in them in a structured and systematic manner. We, therefore, recommend conducting a follow-up study to investigate the direct acquisition of skill development strategies in pre-service and in-service PE teachers.

## Conclusion

The current study demonstrates that an SEL training program can be successfully implemented in PE classes in elementary schools to enhance their SEL skills. This important finding should be addressed by policymakers in the education system and should be leveraged by teacher training programs and colleges. The topic of SEL in Israeli schools has gained increasing attention in recent years. As a result, increasing related efforts can be seen (Appendix 1), such as teacher training programs conducted by the Mofet Institute and the Ministry of Education for promoting SEL. These initiatives strive to provide educators, including kindergarten teachers, with the necessary know-how and abilities for helping youngsters develop their social and emotional skills. One such example can be seen in the Education for Sustainable Development Program, which includes SEL components and offers resources and strategies for integrating SEL principles into teaching applications that can be used in pre-school and school frameworks.

## Data Availability

The original contributions presented in the study are included in the article/Supplementary material, further inquiries can be directed to the corresponding author.

## Appendix 1

SEL Teacher Training Programs and PE Curricula

Avney Rosha, the Israeli Institute for School Leadership, SEL – Knowledge pool for school leadership. [Hebrew] https://avneyrosha.org.il/resourcecenter/Pages/sel.aspx

Central School for Teacher’s Continued Education. SEL curricula. [Hebrew] https://www.dmag.co.il/pub/histadrut/hishtalmut22/28/

Ministry of Education, Curriculum for physical education, for grades 3–6. [Hebrew] https://meyda.education.gov.il/files/Curriculum/third/027055.pdf

Ministry of Education, Pedagogical Administration. The role and I: Workshop for educational teams on the topic of reflection on SEL processes. [Hebrew] https://meyda.education.gov.il/files/yesodi/Hevra/htafkid_veani_sdna.pdf

Mofet Institution. SEL-based management – for beginners. [Hebrew]. https://store-stage.macam.ac.il/store/workshops/10526/

Oranim Academic College and Teaching, Social and Emotional Learning course. [Hebrew] https://www.oranim.ac.il/sites/heb/academic-units/advanced-studies-faculty/kidum/professional/sel/pages/default.aspx

Pisga Lod, Professional Development in the 21^st^ century: Education, digitalization, and multi-cultural. [Hebrew] https://pisga.tik-tak.co.il/webPro/hishtalmuyot/indexHish/lessons.asp?codeClient=2179&id=113363&swviewindex=2

Uriel training center for council facilitators (1). Continuing education program on SEL principles. [Hebrew] https://urielcenter.ravpage.co.il/%D7%9C%D7%9E%D7%99%D7%93%D7%94%20%D7%A8%D7%92%D7%A9%D7%99%D7%AA%20%D7%97%D7%91%D7%A8%D7%AA%D7%99%D7%AA

Uriel training center for council facilitators (2). Continuing education program on SEL principles. [Hebrew] https://www.urielcenter.co.il/%D7%9E%D7%A2%D7%92%D7%9C%D7%99-%D7%94%D7%A7%D7%A9%D7%91%D7%94-%D7%91%D7%97%D7%99%D7%A0%D7%95%D7%9A-%D7%95%D7%94%D7%A9%D7%AA%D7%9C%D7%9E%D7%95%D7%99%D7%95%D7%AA/%D7%A2%D7%A7%D7%A8%D7%95%D7%A0%D7%95%D7%AA-%D7%94%D7%9C%D7%9E%D7%99%D7%93%D7%94-%D7%97%D7%91%D7%A8%D7%AA%D7%99%D7%AA-%D7%A8%D7%92%D7%A9%D7%99%D7%AA/
