# Supplementary material for: Social-emotional learning in physical education classes at elementary schools
Source: Front Psychol. 2025 Apr 4;16:1499240. doi: 10.3389/fpsyg.2025.1499240 (PMC12007113; doi:10.3389/fpsyg.2025.1499240)
Supplement: Supplementary file 1 [file Table_1.docx]

**Appendices**

| **Correlations** | | | | | | | | | | | |
| --- | --- | --- | --- | --- | --- | --- | --- | --- | --- | --- | --- |
|  | | | Teamwork 1 | Teamwork2 | Teamwork3 | Self-awareness2 | Self-awareness3 | Self-awareness1 | Creative thinking1 | Creative thinking2 | Creative thinking3 |
| Spearman's rho | Teamwork1 | Correlation Coefficient | 1.000 | .821^**^ | .711^**^ | .784^**^ | .695^**^ | .967^**^ | .870^**^ | .722^**^ | .644^**^ |
|  |  | Sig. (2-tailed) | . | .000 | .000 | .000 | .000 | .000 | .000 | .000 | .000 |
|  |  | N | 185 | 185 | 185 | 185 | 185 | 185 | 185 | 185 | 185 |
|  | Teamwork2 | Correlation Coefficient | .821^**^ | 1.000 | .777^**^ | .931^**^ | .743^**^ | .790^**^ | .718^**^ | .893^**^ | .745^**^ |
|  |  | Sig. (2-tailed) | .000 | . | .000 | .000 | .000 | .000 | .000 | .000 | .000 |
|  |  | N | 185 | 185 | 185 | 185 | 185 | 185 | 185 | 185 | 185 |
|  | Teamwork3 | Correlation Coefficient | .711^**^ | .777^**^ | 1.000 | .732^**^ | .968^**^ | .692^**^ | .633^**^ | .697^**^ | .931^**^ |
|  |  | Sig. (2-tailed) | .000 | .000 | . | .000 | .000 | .000 | .000 | .000 | .000 |
|  |  | N | 185 | 185 | 260 | 185 | 260 | 185 | 185 | 185 | 260 |
|  | Self-awareness2 | Correlation Coefficient | .784^**^ | .931^**^ | .732^**^ | 1.000 | .796^**^ | .777^**^ | .696^**^ | .920^**^ | .748^**^ |
|  |  | Sig. (2-tailed) | .000 | .000 | .000 | . | .000 | .000 | .000 | .000 | .000 |
|  |  | N | 185 | 185 | 185 | 185 | 185 | 185 | 185 | 185 | 185 |
|  | Self-awareness3 | Correlation Coefficient | .695^**^ | .743^**^ | .968^**^ | .796^**^ | 1.000 | .682^**^ | .624^**^ | .721^**^ | .945^**^ |
|  |  | Sig. (2-tailed) | .000 | .000 | .000 | .000 | . | .000 | .000 | .000 | .000 |
|  |  | N | 185 | 185 | 260 | 185 | 260 | 185 | 185 | 185 | 260 |
|  | Self-awareness1 | Correlation Coefficient | .967^**^ | .790^**^ | .692^**^ | .777^**^ | .682^**^ | 1.000 | .904^**^ | .712^**^ | .636^**^ |
|  |  | Sig. (2-tailed) | .000 | .000 | .000 | .000 | .000 | . | .000 | .000 | .000 |
|  |  | N | 185 | 185 | 185 | 185 | 185 | 185 | 185 | 185 | 185 |
|  | Creative thinking1 | Correlation Coefficient | .870^**^ | .718^**^ | .633^**^ | .696^**^ | .624^**^ | .904^**^ | 1.000 | .696^**^ | .614^**^ |
|  |  | Sig. (2-tailed) | .000 | .000 | .000 | .000 | .000 | .000 | . | .000 | .000 |
|  |  | N | 185 | 185 | 185 | 185 | 185 | 185 | 185 | 185 | 185 |
|  | Creative thinking2 | Correlation Coefficient | .722^**^ | .893^**^ | .697^**^ | .920^**^ | .721^**^ | .712^**^ | .696^**^ | 1.000 | .804^**^ |
|  |  | Sig. (2-tailed) | .000 | .000 | .000 | .000 | .000 | .000 | .000 | . | .000 |
|  |  | N | 185 | 185 | 185 | 185 | 185 | 185 | 185 | 185 | 185 |
|  | Creative thinking3 | Correlation Coefficient | .644^**^ | .745^**^ | .931^**^ | .748^**^ | .945^**^ | .636^**^ | .614^**^ | .804^**^ | 1.000 |
|  |  | Sig. (2-tailed) | .000 | .000 | .000 | .000 | .000 | .000 | .000 | .000 | . |
|  |  | N | 185 | 185 | 260 | 185 | 260 | 185 | 185 | 185 | 260 |
| **. Correlation is significant at the 0.01 level (2-tailed). | | | | | | | | | | | |

| **Correlations** | | | | | | | | | | |
| --- | --- | --- | --- | --- | --- | --- | --- | --- | --- | --- |
|  | | Teamwork 1 | Teamwork2 | Teamwork3 | Self-awareness2 | Self-awareness3 | Self-awareness1 | Creative thinking1 | Creative thinking2 | Creative thinking3 |
| Teamwork1 | Pearson Correlation | 1 | .803^**^ | .771^**^ | .782^**^ | .739^**^ | .979^**^ | .889^**^ | .706^**^ | .668^**^ |
|  | Sig. (2-tailed) |  | .000 | .000 | .000 | .000 | .000 | .000 | .000 | .000 |
|  | N | 185 | 185 | 185 | 185 | 185 | 185 | 185 | 185 | 185 |
| Teamwork2 | Pearson Correlation | .803^**^ | 1 | .783^**^ | .910^**^ | .748^**^ | .783^**^ | .704^**^ | .834^**^ | .716^**^ |
|  | Sig. (2-tailed) | .000 |  | .000 | .000 | .000 | .000 | .000 | .000 | .000 |
|  | N | 185 | 185 | 185 | 185 | 185 | 185 | 185 | 185 | 185 |
| Teamwork3 | Pearson Correlation | .771^**^ | .783^**^ | 1 | .711^**^ | .953^**^ | .750^**^ | .677^**^ | .638^**^ | .913^**^ |
|  | Sig. (2-tailed) | .000 | .000 |  | .000 | .000 | .000 | .000 | .000 | .000 |
|  | N | 185 | 185 | 260 | 185 | 260 | 185 | 185 | 185 | 260 |
| Self-awareness2 | Pearson Correlation | .782^**^ | .910^**^ | .711^**^ | 1 | .803^**^ | .777^**^ | .692^**^ | .911^**^ | .754^**^ |
|  | Sig. (2-tailed) | .000 | .000 | .000 |  | .000 | .000 | .000 | .000 | .000 |
|  | N | 185 | 185 | 185 | 185 | 185 | 185 | 185 | 185 | 185 |
| Self-awareness3 | Pearson Correlation | .739^**^ | .748^**^ | .953^**^ | .803^**^ | 1 | .728^**^ | .648^**^ | .718^**^ | .948^**^ |
|  | Sig. (2-tailed) | .000 | .000 | .000 | .000 |  | .000 | .000 | .000 | .000 |
|  | N | 185 | 185 | 260 | 185 | 260 | 185 | 185 | 185 | 260 |
| Self-awareness1 | Pearson Correlation | .979^**^ | .783^**^ | .750^**^ | .777^**^ | .728^**^ | 1 | .912^**^ | .697^**^ | .660^**^ |
|  | Sig. (2-tailed) | .000 | .000 | .000 | .000 | .000 |  | .000 | .000 | .000 |
|  | N | 185 | 185 | 185 | 185 | 185 | 185 | 185 | 185 | 185 |
| Creative thinking1 | Pearson Correlation | .889^**^ | .704^**^ | .677^**^ | .692^**^ | .648^**^ | .912^**^ | 1 | .690^**^ | .630^**^ |
|  | Sig. (2-tailed) | .000 | .000 | .000 | .000 | .000 | .000 |  | .000 | .000 |
|  | N | 185 | 185 | 185 | 185 | 185 | 185 | 185 | 185 | 185 |
| Creative thinking2 | Pearson Correlation | .706^**^ | .834^**^ | .638^**^ | .911^**^ | .718^**^ | .697^**^ | .690^**^ | 1 | .805^**^ |
|  | Sig. (2-tailed) | .000 | .000 | .000 | .000 | .000 | .000 | .000 |  | .000 |
|  | N | 185 | 185 | 185 | 185 | 185 | 185 | 185 | 185 | 185 |
| Creative thinking3 | Pearson Correlation | .668^**^ | .716^**^ | .913^**^ | .754^**^ | .948^**^ | .660^**^ | .630^**^ | .805^**^ | 1 |
|  | Sig. (2-tailed) | .000 | .000 | .000 | .000 | .000 | .000 | .000 | .000 |  |
|  | N | 185 | 185 | 260 | 185 | 260 | 185 | 185 | 185 | 260 |
| **. Correlation is significant at the 0.01 level (2-tailed). | | | | | | | | | | |
