# Supplementary material for: Social-emotional learning in physical education classes at elementary schools
Source: Front Psychol. 2025 Apr 4;16:1499240. doi: 10.3389/fpsyg.2025.1499240 (PMC12007113; doi:10.3389/fpsyg.2025.1499240)
Supplement: Supplementary file 2 [file Table_2.docx]

**Appendix A -** **Observation of skill assessment indicators during activity:**

**Date:** ____________ **Time:** ____________ **School name:** ____________ **Class:** ____________**Teacher's name:** ____________ **Number of participants:** ____________ **When did the program start?** ____________

|  | **Starting point** | **Started the journey** | **Midway through the journey** | **Reached the destination** |
| --- | --- | --- | --- | --- |
| **Teamwork - Social** |  |  |  |  |
| **Promoting a supportive and non-judgmental Climate** | Does not show respect  Uses tone, body language, and facial expressions that convey a negative attitude towards team members.  Shows contempt for the team or the task  Does not encourage team members  Works alone, ignores the group | Sometimes shows respect and sometimes contempt.  Sometimes works alone and sometimes in a team.  His body language is sometimes respectful and sometimes contemptuous towards team members.  Some tasks are treated with contempt, some with respect. | Encourages a supportive climate to some extent:  Shows respect to some team members  Sometimes uses positive body language  Sometimes motivates team members to act  Sometimes helps and promotes team members | Encourages a supportive climate by consistently performing all of the following actions:  Shows respect to team members  Uses positive body language and conveys a positive attitude  Motivates team members to act  Confident in the success of the group |
| **Respond to conflicts among team members** | Tends to get angry or argue | Sometimes still gets angry, and sometimes manages to restrain | Neutral to different viewpoints | Actively finds a solution to the conflict to strengthen the group |
| **Communication Culture** | Does not respond to team members' words | Sometimes responds to team members' words and sometimes does not respond or participate in turn | Participates in turn and listens to others \| Encourages team members' contributions and builds on ideas | Ensures all team members participate |
| **Punctuality and following instructions** | Does not meet deadlines and does not follow instructions | Sometimes meets deadlines and sometimes follows instructions | Usually meets deadlines and usually follows instructions | Fully meets deadlines and follows instructions |
| **Contribution to the group's success in the task** | Does not share own ideas to promote task development | Sometimes shares ideas, and sometimes does not | Shares own ideas but does not consider others' ideas | Shares own ideas and offers alternative solutions based on others' ideas |
| **Emotional - self-awareness** |  |  |  |  |
| Perseverance in the Face of Challenges | Does not persist in task execution and stops without reaching the goal | Sometimes persists, and sometimes gives up | Persists in the face of difficulty and tries different ways but does not reach the goal | Students succeed in setting goals and strive to achieve them, reaching the goal. |
| **Internal locus of control**  **Reflective ability** | Does not know how to express and describe feelings and emotions  How was it for me?  What did I enjoy?  What did I like less?  How was the group work for me?  Students fail to think reflectively and do not recognize the connection between action and result. | Partially know how to express feelings and emotions following work | Recognizes strengths and challenges  What am I good at?  Where was it challenging for me?  What can I improve?  What can the group improve?  Students succeed in conducting a reflective process of what works and what does not but still do not see the connection between action and result. | Recognizes the group’s strengths and challenges  What did I learn from the group?  What is my contribution to the group?  Students succeed in thinking reflectively about the process and seeing the connection between action and results. |
| **Perseverance in the face of difficulties and failures** | Do not cope, give up, get frustrated, and raise hands. | Sometimes, copes, and sometimes gives up.  Sometimes, tries, but only a few times. | Attempts several times but still fails and ultimately gives up. | Continue trying and eventually solve in several different ways |
| **Proactivity** | Students are not active and avoid continuing and trying | Sometimes, students are active and sometimes not | Students work diligently and succeed in the task | Students challenge themselves at a high level of difficulty, initiate new challenges, help others. |
| **Creative thinking - generic cognitive** |  |  |  |  |
| Innovation and acquisition of strategies and skills | Duplicate, copy, or repeat existing ideas | Sometimes, they still copy, and sometimes, they succeed in thinking of a new idea of their own | Adapt or adjust ideas into a new product | Create entirely new ideas themselves  Change and transform ideas into new forms or dimensions |
| **Problem solving and taking risks** | Strictly adhere to instructions. | Sometimes they still adhere to instructions and sometimes succeed in thinking of one new direction | Consider new directions or new approaches to solving the problem | Take new risks that have not yet been chosen. Develop an original way to solve the problem and can express the reasons for the choice |

**Sample questions for a semi-structured interview with the teacher:**

1. Have you noticed any changes in the children following the program?
2. What can you say about the level of cooperation among the children? (Asking questions in class, showing interest and curiosity, completing tasks)
3. How do you experience the atmosphere among the students during the activity?
4. Describe the conversation/atmosphere among the students.
5. What teamwork skills do you identify that the students are acquiring?
6. What is your opinion regarding the students' ability to succeed in tasks?
7. How do you think the students cope with challenges and difficulties?
8. How do you assess the students' reflective skills during the activity? How is this manifested?
9. What ways of thinking do you think the students employ during the activity?
10. Would you change anything in the program to better achieve the goals or to achieve additional goals?
